# Complete Genome Sequencing of Polar *Arthrobacter* sp. PAMC25284, Copper Tolerance Potential Unraveled with Genomic Analysis

**DOI:** 10.1155/2022/1162938

**Published:** 2022-08-25

**Authors:** Jayram Karmacharya, Prasansah Shrestha, So-Ra Han, Hyun Park, Tae-Jin Oh

**Affiliations:** ^1^Department of Life Science and Biochemical Engineering, Graduate School, SunMoon University, Asan 31460, Republic of Korea; ^2^Division of Biotechnology, College of Life Sciences and Biotechnology, Korea University, Seoul 02841, Republic of Korea; ^3^Genome-based BioIT Convergence Institute, Asan 31460, Republic of Korea; ^4^Department of Pharmaceutical Engineering and Biotechnology, SunMoon University, Asan 31460, Republic of Korea

## Abstract

The genus *Arthrobacter* is a known group of Gram-positive, opportunistic pathogenic bacteria from cold climates, with members that are believed to play a variety of roles at low temperatures. However, their survival mechanisms in frigid environments like the Antarctic are still unknown. We identified a species of *Arthrobacter* isolated from seawater in the polar region using 16S rRNA sequence analysis. The strain PAMC25284 genome consists of a circular chromosome with a GC content of 65.6% and is projected to contain 3,588 genes, of which 3,150 are protein coding, 366 are pseudogenes, 19 are rRNA coding, and 50 are tRNA coding genes. Using comparative genomics, we showed that PMAC25284 has copper-transporting ATPases, copper chaperone, copper-responsive transcriptional regulator, and multi-copper oxidase domains, which are found in both Gram-positive (like *Mycobacterium tuberculosis* and *Enterococcus hirae*) and Gram-negative bacteria (like *E*. *coli* and *Pseudomonas aeruginosa*). The existence of 4 multi-copper oxidase genes, which supplied an additional copper defense mechanism, could be intriguing information regarding Gram-positive bacteria such as *Arthrobacter* sp. PAMC25284. In addition, our strain PAMC25284 has the same *MmcO* gene as *M. tuberculosis*, with a locus tag KY499_RS04055 similarity of 40.61%, which is the highest among the Gram-positive and Gram-negative bacteria studied for this gene. Our cold-adapted *Arthrobacter* sp. strain PAMC25564 was published previously but did not contain a multi-copper oxidase domain-containing gene, but strain PAMC25284 was studied in this study.

## 1. Introduction

Glacier habitats have rich and diverse microbial communities with unique adaptive characteristics. Among such cold inhabitants, *Actinobacteria* with high GC content are considered to be the most common [1]. The phylum Actinobacteria is made up of phylogenetically diverse organisms that have been studied for their ability to cause diseases in plants and animals, produce antimicrobial compounds and antitumor agents, and degrade recalcitrant molecules in soil environments [2]. Within *Actinobacteria*, members of the genus *Arthrobacter* are notable because they are among the most frequently found in soil environments. Their widespread distribution is due to their dietary versatility and tolerance to environmental challenges. Compared to mesophilic *Arthrobacter* isolates, Antarctic *Arthrobacter* strains showed genome content scaling as an adaptation alteration, exhibiting fewer protein-coding sequences and a lower number of transcription and carbohydrate metabolism-associated genes [1]. Ubiquitous organisms are assumed to have key roles in the biogeochemistry of heavy metals due to their fundamental features as bioconverters [3]. As a result, studying the responses of microbes to metals is of scientific interest and may be useful in developing biotechnological solutions for the recovery and purification of important and/or harmful metals in the environment.

Copper is a metal ion that has been shown to be hazardous to bacteria and other organisms. Excess copper, regardless of its valence state, binds to a wide range of biomolecules, including proteins, lipids, and nucleic acids [4]. However, unlike other poisonous metals, like silver and lead, copper is also an important trace nutrient. Bacteria developed strict copper homeostatic control systems involving copper binding and transport, as well as copper-mediated gene regulation. The copper resistance is encoded by the *cop* genes (*copA, copB, copC, copD, copY*, and *copZ*) in *Cupriavidus metallidurans* CH34, *P. aeruginosa* PAO1, and *E. hirae* and by the *pco* genes (*pcoA, pcoB, pcoC*, and *pcoD*) in *Escherichia coli* [5, 6]. CopA and CopB are copper-transporting ATPases, while CopY is a copper-responsive repressor, and CopZ is a chaperone that catalyzes intracellular copper routing [6]. The *copA* gene encoding a multi-copper oxidase (*pcoA* gene in *E. coli*) is one of the main genetic determinants involved in Cu resistance in Gram-negative bacteria. In *E. hirae*, this copper can participate in the metalation of cuproenzymes in some rare cases. Copper defense by multi-copper oxidase has been reported in various bacteria, including *Campylobacter jejuni*, *Myxococcus xanthus*, *Rhodobacter capsulatus*, *Salmonella enterica*, *Staphylococcus aureus*, and *Xanthomonas campestris*, in addition to *E. coli* [7]. In nature, multi-copper oxidase functionality varies depending on the source organism and the surroundings. Laccases (EC 1.10.3.2) and a broad family of copper oxidases, such as ascorbate oxidases (EC 1.10.3.3), ceruloplasmin (EC 1.16.3.1), bilirubin oxidase (EC 1.10.3.4), and metallo-oxidases Fet3p (EC 1.16.1.3.1), are all included in multi-copper oxidase [8]. In bacteria, they play important roles in spore coat resistance [9], melanin production [10], morphogenesis [11], metal oxidation [12], and denitrification [13]; in fungi, pigment formation [14], lignin degradation [15], dissimilatory nitrite reduction [16], and virulence [17]; in yeasts, iron uptake [18]; in insects, cuticle tanning [19]; in plants, lignin biosynthesis and ascorbate metabolism [20]; in mammals, iron metabolism [21].

In this study, we performed comprehensive genome sequencing on *Arthrobacter* sp. PAMC25284, a psychrotolerant bacterium originally isolated from seawater collected from the South Shetland Islands, Barton Peninsula, Antarctica. Various genes involved with copper resistance are highlighted herein. To our knowledge, this is the first study to provide genetic and phenotypic insight into *Arthrobacter* sp. PAMC25284 derived from Antarctica seawater and its potential role in copper resistance.

## 2. Materials and Methods

### 2.1. Taxonomic Identification

The *Arthrobacter* sp. PAMC25284 was isolated from the seawater of the South Shetland Islands, Barton Peninsula (62°13.536′ S; 58°47.054′ W) using 0.1X RA agar (MB cell Ltd., Seoul, Korea), and it was acquired at the environmental temperature of 20°C. The bacterial sample for DNA analysis was done at 15°C temperature using pure R2A agar media. The DNA from strain PAMC25284 was extracted using a QIAmp DNA Mini Kit (Qiagen Inc., Valencia, CA, USA). Genome quality and concentration were determined by a spectrophotometer (Biochrome, Libra S35PC, UK) and detected by agarose gel electrophoresis to evaluate its quality.

### 2.2. Complete Genome Sequencing and Annotation

Genome sequencing was performed using PacBio sequel single-molecule real-time (SMRT) sequencing technology (Pacific Biosciences, Menlo Park, CA, USA). SMRTbell library inserts (20 kb) were sequenced using SMRT cells. Raw sequence data were generated from 77,075 reads and 821,081,934 bp that were assembled *de novo* using the hierarchical genome assembly process (HGAP) protocol [22] and HGAP4 assembly using SMRT analysis software (ver. 2.3; Pacific Biosciences, https://github.com/PacificBiosciences/SMRT-Analysis). The complete genome sequence was deposited in the GenBank database under the GenBank accession number NZ_CP080382.1 (Bio project number PRJNA748195).

The PAMC25284 genome was annotated using the rapid annotation subsystem technology (RAST) server [23]. The predicted gene sequences were translated and searched in the National Center for Biotechnology Information (NCBI) nonredundant database, the Cluster of Orthologous Groups (COG) from the eggnog v.4.5.1 database [24], and the Kyoto Encyclopedia of Genes and Genomes (KEGG) database. A circular map of the PAMC25284 genome was prepared using the CGView^BETA^ comparison tool [25].

### 2.3. Genotypic Analysis of Arthrobacter sp. PAMC25284

The phylogenetic analysis of the *Arthrobacter* sp. PAMC25284 16s rRNA gene sequence and the sequences of the type strains of the species within the family Micrococcaceae was performed using MEGAX software [26], based on the alignment of the sequence with ClustalW [27]. The distances were calculated using Kimura's two-parameter model [28], and the phylogenetic tree was inferred using maximum likelihood [29] neighbor joining [30] analysis. For phylogenetic tree construction, complete genome sequences of the 16s rRNA and sequences of related type strains were obtained from the EzBioCloud database (http://www.ezbiocloud.net/) [31]. The average nucleotide identity (ANI) values between the genome sequence of strain PAMC25284 and the type strains of the closest related species were estimated using the ANI calculator in the EzBioCloud. The G+C mol.% content of DNA was determined from the complete sequence.

For protein phylogenetic tree construction, the secondary data were used to identify type I copper center protein and its variants. The multi-copper oxidase domain containing the protein sequence of the strain PAMC25284 and type I copper center protein of the related strains were obtained from the UniPort (https://www.uniprot.org/) [32] and NCBI database (https://www.ncbi.nlm.nih.gov/) [33], respectively. The sequences were aligned by MUSCLE [34, 35], and a phylogenetic tree was inferred using maximum likelihood and neighbor joining analysis. The multiple sequence alignment of the related proteins and then identification of the conserved region were performed using Clustal Omega (https://www.ebi.ac.uk/Tools/msa/clustalo/) [36]. Signal IP 5.0 [37] (neural networks and Markov models) and TMHMM 2.0 Server [38] were used to predict the subcellular localization of strain PAMC25284 multi-copper oxidase domain-containing proteins.

### 2.4. Prediction of Cu-Specific Transporters, Chaperones, and Cuproproteins of Arthrobacter sp. PAMCC25284

Cuproproteins, chaperons, and copper-specific transporters of a given organism were predicted using previous literature, protein-protein blast (blastp) search [39], and the highest homology sequences were determined. The sequences were retrieved from the RAST database and BLAST was done against the amino acid sequences to obtain the highest homology sequences.

## 3. Results and Discussion

### 3.1. Complete Genome Profile of Arthrobacter sp. PAMC25284

**Figure 1:**
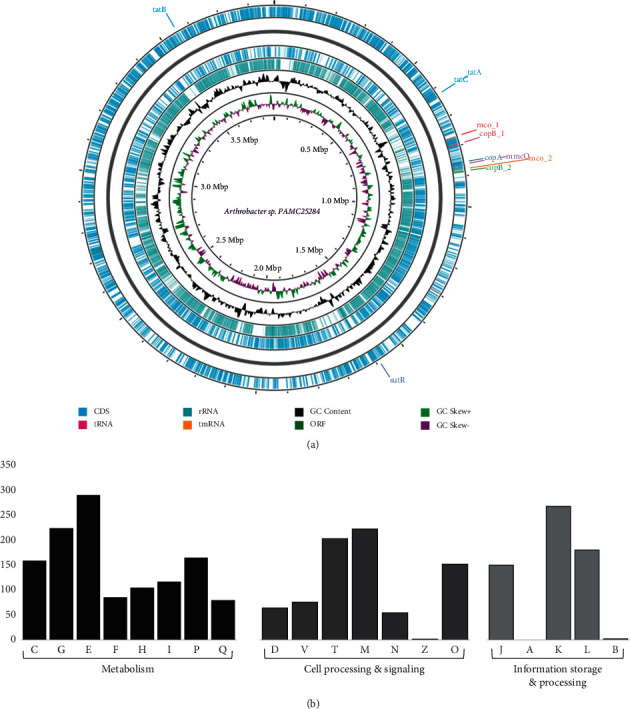
(a) Circular representation of genome and features of the *Arthrobacter* sp. PAMC25284. The contents of the featured rings (starting with the outermost ring to the center) are as follows: Ring 1, CDS (including tRNA and rRNA0 and Pokka annotation with genes that are involved in copper homeostasis; Ring 2, combined ORFs in forward and reverse strands; Ring 3, plot of GC content; Ring 4, GC skew plot, values above average are depicted in green, and below average in purple; and Ring 5, sequence ruler. (b) COG functional categories for forward coding sequences. Metabolism: C, energy production and conversion; G, carbohydrate transport and metabolism; E, amino acid transport and metabolism; F, nucleotide transport and metabolism; H, coenzyme transport and metabolism; I, lipid transport and metabolism; P, inorganic ion transport and metabolism; Q, secondary metabolites biosynthesis, transport, and catabolism. Cell processing and signaling: D, cell cycle control, cell division, and chromosome partitioning; V, defense mechanisms; T, signal transduction mechanisms; M, cell wall/membrane/envelope biogenesis; N, cell motility; Z, mobilome, prophages, and transposons; O, posttranslational modification, protein turnover, and chaperones. Information storage and processing: J, translation, ribosomal structure, and biogenesis; A, RNA processing and modification; K, transcription; L, replication, recombination, and repair; B, chromatin structure and dynamics.

The complete genome of *Arthrobacter* sp. PAMC25284 is comprised of a circular chromosome of 3,883,680 bp with a GC content of 65.6 percent, as shown in Table 1. On the chromosome, 3,588 genes were predicted, with 3,150 protein-encoding genes functionally assigned and the remaining genes predicted as hypothetical proteins. We annotated 366 pseudogenes, 19 rRNA genes, and 50 tRNA genes distributed throughout the genome. Of the predicted genes, 2,860 (90.80%) were classified into 20 functional Clusters of Orthologous Groups (COG) categories, whereas the remaining 290 (9.20%) genes were unclassified. The most numerous COG categories were genes with S genes with unknown function (581 genes), E (289 genes), K (263 genes), and G (223 genes) (Figure 1). Many of these genes are related to amino acid transport, carbohydrate transport, and energy production/conversion. The metabolic flexibility of this strain was discovered through phenotypic assessment of carbon utilization profiles. Furthermore, significantly fewer coding sequences (CDSs) were allocated to the COG categories of transcription [K] and carbohydrate transport and metabolism [G] out of the total CDSs discovered in the genome. In four Antarctic *Arthrobacter* isolates, fewer CDSs, decreased metabolic flexibility, and a significant drop in CDS related to transcription, carbohydrate transport, and metabolism suggest genome content scaling [2].

With further gene subsystem clustering analysis, *Arthrobacter* sp. PAMC25284 with SEED viewer of RAST database showed functional genes with the presence of a total of 279 (26% of the strain's genome) subsystems [40]. The top five subsystems belonged to carbohydrate metabolism (347); amino acids and derivatives (306); protein metabolism (163); cofactors, vitamins, prosthetic groups, and pigments (140); and nucleosides and nucleotides (83). Additionally, functions related to membrane transport (46); stress response (36); resistance to antibiotics and toxic compounds (31); the metabolism of aromatic compounds (16) were also identified. Collectively, these analyses of cold-adapted *Arthrobacter* sp. PAMC25284 suggest the presence of several genome-enabled metabolic and catabolic processes, which might play a significant role in the colonization and its survival in such psychrophilic environments. Similar findings have been reported in psychrophilic *Cryobacterium* species [41], where specific genes in the categories like carbohydrates, cofactors, vitamins, prosthetic groups, pigments, and ABC transporters in membrane transport were predominant.

### 3.2. Phylogenomic Analysis Based on 16S rRNA and Multi-Copper Oxidase Domains

**Figure 2:**
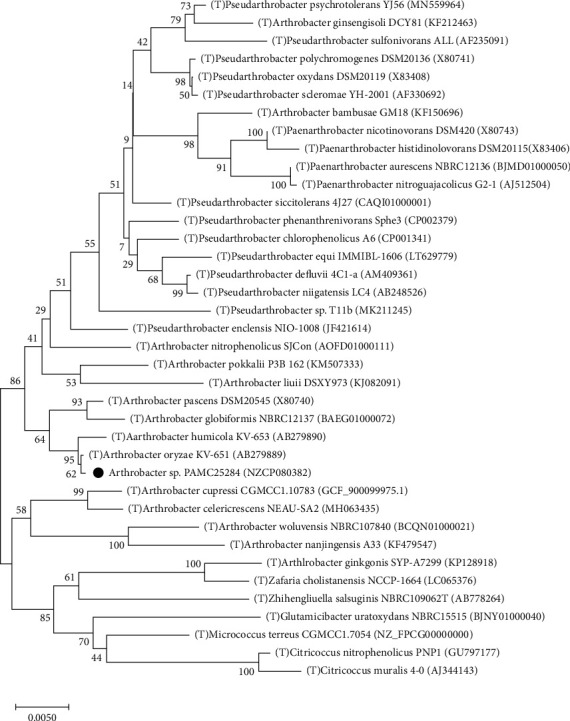
Neighbor joining phylogenetic tree based on 16S rRNA gene sequences showing the relationships between strain PAMC25284 (indicated with a red circle) and the type strains T of related *Arthrobacter* species. The numbers at the nodes indicate the level of bootstrap support based on a maximum likelihood of 1,000 resampled datasets. Scale bar = 0.005 substitutions per nucleotide position. Accession numbers of the sequences are indicated in parentheses.

A phylogenetic tree was constructed based on 16S rRNA sequences that are related to the genus *Arthrobacter* strains, which include strain PAMC25284 (Figure 2). The *Arthrobacter* sp. PAMC25284 shared the same clade with *A. oryzae* KV-651^T^ with 62% bootstrap support and *A. humicola* KV-653^T^ with 95% bootstrap support. According to the EzBioCloud database, five *Arthrobacter* species (*A. oryzae* KV-651^T^, *A. humicola* KV-653^T^, *A. pascens* DSM20545^T^, *A. globiformis* NBRC12137^T^, and *Pseudarthrobacter siccitolerans* 4J27^T^) showed a higher 16S rRNA sequence identity that is more than 98.27%. When comparing type strains, we found that *Arthrobacter* sp. PAMC25284 had ANI values higher than 95%, which is the algorithmic cut-off for species-level identification. *A. oryzae* KV-651^T^ showed the highest ANI value of 99.75% (ANI coverage of 97.16%) with 16S rRNA gene (obtained from EzBioCloud) identity of 99.79%. *A. humicola* KV-653^T^ showed the second highest 16S rRNA sequence identity (99.45%), which was obtained from the EzBioCloud with an ANI value of 99.2%.  Table S1 of the Supplementary Materials (SM) lists details of the database search to identify the strain. As a result, the phylogenetic tree analysis and ANI values revealed the same clade, with the *Arthrobacter* sp. PAMC25284 having the closest relationship to *A. oryzae* KV-651^T^ and *A. humicola* KV-653^T^.

**Figure 3:**
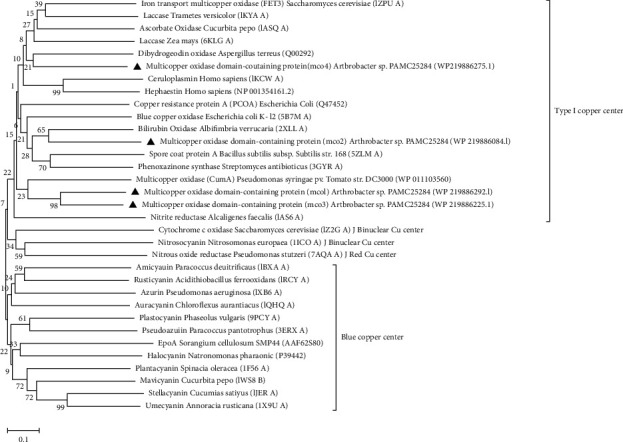
Neighbor joining phylogenetic tree based on multi-copper oxidase domain-containing protein sequences (indicated in triangles) of strain PAMC25284 and type I copper center protein gene sequences. The numbers at the nodes indicate the level of bootstrap support based on a maximum likelihood of 1,000 resampled datasets. Scale bar = 0.10 substitutions per nucleotide position. Accession numbers or protein structure numbers of the sequences are indicated in parentheses.

Based on types of copper in proteins classified as type I copper, type II copper, and type III copper using secondary data [42]. Type I copper comprises a blue copper center, red Cu center, binuclear copper center, and type I copper center [42]. The twenty-nine sequences that are characterized as blue copper centers, type I copper, and variants from different origins along with different biological roles were used for the query. The phylogenetic tree was constructed using neighbor joining alignment revealed that the strain PMAC25284 had a multi-copper oxidase domain-containing protein (as coded mco1, mco2, mco3, and mco4) clusters with different variants of type I copper centers, which is shown in Figure 3. It was found that mco1 and mco3 of strain PAMC25284 were out-border clustered with multi-copper oxidase, CumA of *P. syringae pv. tomato* DC3000 (23% bootstrap support), whereas *mco2* shared the same clade with bilirubin oxidase of *Albifimbria verrucaria* (65% bootstrap support). Moreover, it was revealed that *mco4* of strain PAMC25284 shared the same clade with dihydroquinoline oxidase of *Aspergillus terreus* (21% bootstrap support). Even though multi-copper oxidase domain-containing genes were found in both Gram-positive and Gram-negative bacteria, another strain of *Arthrobacter* sp. PAMC25564 isolated from cryoconite [43] did not have any multi-copper oxidase domain-containing genes. Therefore, this study suggested that even if microorganisms are isolated from the same polar region, their functional genes differ depending on their habitat.

### 3.3. Multicopper Oxidase Sequence Analysis

**Figure 4:**
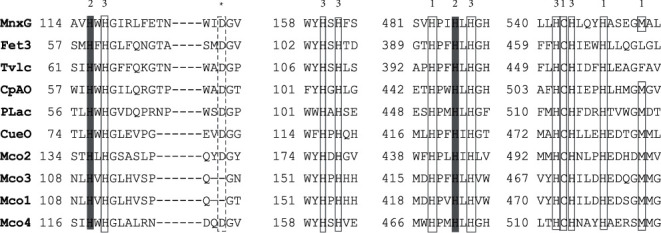
Homology of amino acid sequence around the copper-binding sites of type I Cu center and multi-copper oxidases of the strain *Arthrobacter* sp. PAMC25284. 1, 2, 3, and asterisk denote type I copper ligand, type II copper ligand, type III copper ligand, and proton donor, respectively. MnxG, dihydrogeodin oxide; Fet3, iron transport multi-copper oxidase; Tylc, *Trametes* versicolor laccase; CpAO, *Cucurbita pepo* ascorbate oxidase; Plac, *Zea mays* laccase; CueO, blue copper oxidase; and Mco1–Mco4, *Arthrobacter* sp. PAMC25284 multi-copper oxidase domain-containing proteins.

**Figure 5:**
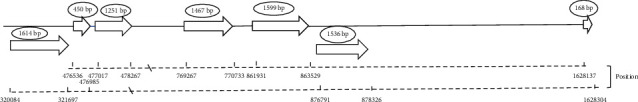
The gene clusters of multi-copper oxidase domain within the genome of *Arthrobacter* sp. PAMC25284, based on annotations in the SEED database. The arrowheads represent the genes with a start and stop position in the chromosome, along with their respective lengths.

The protein sequences of only six different strains were extracted as a model containing the residues considered significant in copper coordination, along with multi-copper oxidase domains incorporating protein sequences from the strain PAMC25284 (Figure 4), where the typical hallmarks of all MCOs were identified. The ligand groups commonly coordinating the type I copper center in MCO are 1Cys and 2His residues [42]. MCO protein is identified by the presence of highly conserved histidine and cytosine rich signature sequences (HXHG, HXH, HXXHXH, and HCHXXXHXXXXM/L/F) inside the cupredoxin domain [44]. The bioinformatics tools like Signal P 5.0 predicted all multi-copper oxidase domain-containing proteins of strain PAMC25284 are intracellular, whereas the TMHMM Server 2.0 predicted a transmembrane domain in the C-terminal region of all multi-copper oxidase domain-containing proteins [38]. The genome of *Arthrobacter* sp. PAMC25284 possessed four multi-copper oxidase domain-containing gene clusters in downstream (Figure 5 and Table S2 of the Supplementary Materials), which have not been reported in *Arthrobacter* until now. Therefore, this study might be the first to report the occurrence of four multi-copper oxidase domain-containing genes in the genus *Arthrobacter*.

### 3.4. Distribution of Cu Transporter Systems and Multi-Copper Oxidases

The strain PAMC25284 comprises the Cu importers/exporters and multi-copper oxidase in comparison with strains of Gram-positive (*E. hirae* ATCC9790 and *M. tuberculosis* H37Rv) and Gram-negative (*P. aeruginosa* PAO1, *E. coli* DH5*α*, and *P. fluorescens* SBW25) bacteria, as shown in Table 2. Copper is a necessary metal for the self-regulating processes of plants, bacteria, and eukaryotic organisms, as it can easily bind with high affinity to a variety of proteins due to its transition states, i.e., reduced (Cu^+^) and oxidized (Cu^2+^) with low-energy barrier [45]. The affinity residues are the thiol and thioether groups of cysteine/methionine and imidazole groups of aspartic/glutamic acid or histidine, which determine the protein structural states and functions in biological systems and cause possible toxic effects. Copper-dependent proteins involved in copper homeostasis are transcriptional regulators, chaperones and storage proteins, cell surface/secretory transporters and receptors, oxidoreductases, electron transfer/energy production/blue Cu proteins, free radical scavenging protein, oxidase, and monooxygenases [46].

**Figure 6:**
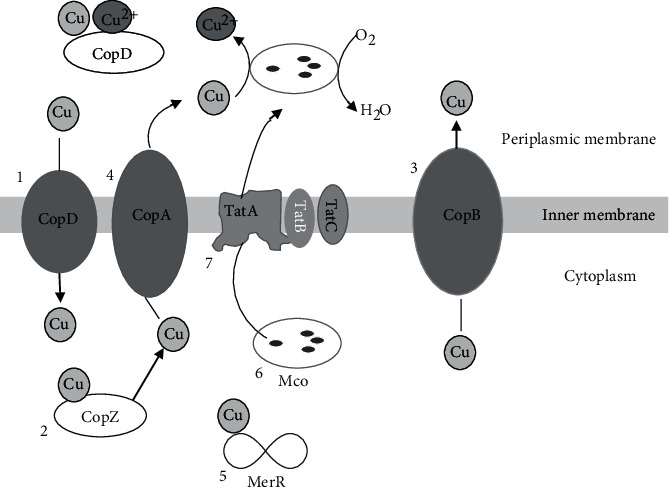
Proteins involved in the copper homeostasis of *Arthrobacter* sp. PAMC25284. The proteins are depicted in the schematic. The proteins are labeled with their name. The information is denoted as follows: 1, copper entry into the cytoplasm; 2, copper sequestration by metallochaperones; 3 and 4, copper secretion by ATPases; 5, cytoplasmic copper complexed with MerR to induce and express CopA copper efflux ATPase, the multi-copper oxidase; 6, MCO/MmcO, multi-copper oxidase domain-containing protein translocated to the periplasm by the twin-arginine translocation system (TatABC), probably as a folded protein.

Three core elements of copper homeostasis are present in both bacteria, Gram-positive and Gram-negative: a copper exporting ATPase (CopA), a copper chaperone (CopZ), and a copper-responsive transcriptional regulator (CopY) [47]. We have found similar core elements of copper homeostasis (CopA, CopZ, and CopY) in the strain PAMC25284, except CopY (Table 2). It is reported that additional defense against copper is provided by the periplasmic CueO-type multi-copper oxidases, which can oxidize Cu ^+^ to less toxic Cu^2+^ and catechol to copper-binding pigments in Gram-negative bacteria, which is intriguing information about Gram-positive bacteria strain PAMC25284 [48–50]. Twin-arginine translocase (Tat) export systems exist in both Gram-positive and Gram-negative bacteria [51], which export proteins across the cytoplasmic membrane in a posttranslational manner. Table S2 of the Supplementary Materials and Figure 6 summarize Tat export systems and other elements, along with the sequence similarity.

### 3.5. Copper Defense Mechanisms in Arthrobacter sp. PAMC25284

Copper transport into the cytoplasm was proposed to be mediated by the transmembrane protein, CopD, which has been characterized in *P. fluorescens* SBW25 [52]. Likewise, strain PAMC25284 also possesses CopD sequence but no similarity with the Gram-negative strains like *P*. *fluorescens* SBW25 and less similarity with *E. coli* DH5*α* (26.37%) (Table S3 of Supplementary Materials). Furthermore, Gram-positive bacteria, such as *E. hirae* ATCC9790 and *M. tuberculosis* H37RV, do not have CopD protein. Therefore, it is possible that our Gram-positive strain PAMC25284 has CopD protein along with CopZ protein. However, it had only 32.26% of its amino acid identity (homology) with Gram-positive strain *E*. *hirae* ATCC9790 and 35.94% with Gram-negative strain *P. aeruginosa* PAO1. Copper entering the cytoplasm is complexed by the CopZ-like copper chaperone, which directs it to regulators of gene expression and the CopA ATPases for export into the periplasmic space [47]. CopA was a member of the P-type ATPases superfamily. The strain PAMC25284 includes five different P-type ATPases (KY499_RS03705, KY499_RS04025, KY499_RS04195, KY499_RS04195, and KY499_RS12155) in different loci (obtained from the NCBI database); it was possible that it had CopA protein because the KY499_RS04025 locus sequence shares 41.66% (the highest) with CopA of *E. hirae.* Even though *E. hirae* ATCC9790 has two P-type ATPases like *P. aeruginosa* PAO1, only CopB had been demonstrated to confer copper tolerance [53]. CopB from *E. hirae* ATCC9790 was the first P-type ATPase whose transport in membrane vesicles was directly demonstrated using ^64^Cu^+^ [54]. CopB of *E. hirae* ATCC9790 shared 43.90% identity with the locus KY499_RS03705 of strain PAMC25284. Similarly, a MerR-type copper-responsive transcriptional activator, CueR, regulated the expression of two genes important for copper homeostasis (CopA copper efflux ATPase and the periplasmic CueO multi-copper oxidase). PAMC25284 also had five MerR-type copper-responsive transcriptional activators (KY499_RS05070, KY499_05080, KY499_RS11345, KY499_RS13545, and KY499_RS13545). Among five MerR types, locus tag KY499_RS13545 found high similarity with CueR of *E. coli* DH5*α*, whereas with CueR of *P. aeruginosa*, PAO1 had the highest similarity with locus tag KY499_RS11345. It has been shown that the connection between CopZ and MerR enhances CopA activation and copper sequestration in the periplasm [55].

The strain PAMC25284 has five multi-copper oxidase domain-containing proteins with varying loci (KY499_RS02210, KY499_RS04160, KY499_RS01525, KY499_RS03595, and KY499_RS04055). CueO of *E. coli* is a multi-copper oxidase that had robust cuprous oxidase activity that could contribute to copper resistance [49, 56]. One possible contribution of CueO to copper tolerance was the oxidation of toxic Cu ^+^ to Cu^2+^ [48, 50]. A second mechanism by which CueO could contribute to copper resistance was by the oxidation of siderophores and other phenolic compounds to their polyphenols [49]. Among them, the highest protein sequence similarity to CueO of *E. coli* DH5*α* was at the locus tag KY499_RS04055 (34.85%). A similar role in copper tolerance was also demonstrated for the MmcO of *M. tuberculosis* H37Rv [57]. MmcO has lipidation at Cys35 and is secreted by the Tat secretion system [50], which indicates that the protein may be membrane associated in the periplasm of *M. tuberculosis*. When compared to the strain PAMC25284 multi-copper oxidase domain-containing protein, the protein with locus tag KY499_RS04055 had the highest similarity of 40.61%. TatA, TatB, and TatC are the three proteins found in the *Arthrobacteria* Tat export system, which was like that of *M. tuberculosis* H37Rv [58–60]. TatA protein of PAMC25284 resembled TatA of *E. coli* DH5*α* by 29.87%, whereas TatA proteins of *P. aeruginosa* PAO1 and *M. tuberculosis* H37Rv by 28.57% and 50%, respectively. Similarly, TatB protein of strain PAMC25284 is similar to TatB of *E. coli* DH5*α* by 33.93%, whereas the TatB proteins of *P. aeruginosa* PAO1 and *M. tuberculosis* H37Rv were similar by 31.58%, and 27.05% respectively. The last subunit TatC protein of PAMC25284 resembled *E. coli* DH5*α* by 33.07%, whereas the TatC proteins of *P. aeruginosa* PAO1 and *M. tuberculosis* H37Rv by 34.39% and 35.64%, respectively. TatA and TatB proteins form a complex that contains the binding site for Tat preproteins [61, 62]. After a preprotein binds to TatBC, TatA protein was recruited to the complex [61]. TatA protein was generally believed to form an export channel and is found in homo-oligomers of varying sizes, which may give the Tat export the flexibility to export folded proteins of different sizes and shapes [63–65].

## 4. Conclusions

In summary, we elucidated the complete genome sequence of polar *Arthrobacter* sp. PMAC25284 and compared it to copper resistance genes characterized by nonpolar Gram-positive and Gram-negative strains. The polar *Arthrobacter* sp. PMAC25284 was isolated from seawater under laboratory conditions and confirmed it by analysis of 16s rRNA sequences. Even though this strain has been previously isolated from harsh and noncontaminated conditions, there are no reports of copper genes being employed in such a cold environment. The genome of *Arthrobacter* sp. PAMC25284 is 3.89 Mb in size and has a GC content of 65.6%, indicating that this strain has high GC content despite its small genomic size. The copper-transporting ATPase, a copper chaperon, and copper-responsive transcriptional regulators associated with copper resistance genes are all described for the first time in *Arthrobacter* species. We confirmed that PMAC25284 has 5 P-type ATPases, 1 TatABC translocation system (TatABC), 1 copper chaperone, 5 transcription factors (MerR), 5 multi-copper oxidase proteins (MCO), and 2 copper uptake systems. Further functional analysis of the identified genes might give insights into the detailed molecular mechanisms of cold-adapted microbes to tolerate and transform copper in copper-contaminated environments. This study provides a foundation to understand how the Gram-positive strain PAMC25284 produces metal-binding molecules to maintain proper metal homeostasis that has allowed bacteria to colonize various extreme environments, like Antarctica.

## Figures and Tables

**Table 1:** Genomic features of *Arthrobacter* sp. PAMC25284.

Features	Value
A: Genomic statistics	
Contings	1
Total length bp	3,883,680
N50	3,883,676
L50	1
GC%	65.6

B: genomic features	
Assembly level	Complete genome
Chromosome genes	3,588
Protein-coding genes	3,150
Pseudogenes	366
rRNA genes	19
tRNA genes	50

**Table 2:** List of cuproproteins and copper transporters, chaperones, and multi-copper oxidases examined in this study of different reference strains with their accession number indicated in parentheses. TF, transcription factor; CU, copper uptake; CYTO-C, cytoplasmic copper chaperone; TatABC, twin-arginine translocation system; *P*-type, *P*-type copper ATPase; MCO, multi-copper oxidase; and —, not applicable.

Bacterial strains	TF	CU	CYTO-C	TatABC	*P*-type	MCO
*Pseudomonas aeruginosa*	CueR	—	CopZ1	—	CopA1	PcoA
PAO1 [NC_002516.2]	—	—	CopZ2	—	CopA2	—
*Escherichia coli*	CueR	CopC	—	TatABC	CopA	PcoA
DH5*α* [NZ_CP026085.1]	—	CopD	—	—	—	CueO
*Pseudomonas fluorescens*	—	CopC	—	—	—	—
SBW25 [NC_012660.1]	—	CopD	—	—	—	—
*Arthrobacter* sp.	SutR	CopC	CopZ	TatABC	CopA	MCO_1
PAMC25284 [NZ_CP080382.1]	—	CopD	—	—	CopB_1	MCO_2
	—	—	—	—	CopB_2	MmcO
*Enterococcus hirae*	—	—	CopZ	—	CopA	NA
ATCC 9790 [NC_018081.1]	—	—	—	—	CopB	—
*Mycobacterium tuberculosis* H37Rv [NC_000962.3]	—	—	—	TatABC	—	MmcO

## Data Availability

The 16S rRNA datasets analyzed during the current study are available in the EzBioCloud repository and NCBI database, accession numbers: MN559964 for *Pseudarthrobacter psychrotolerans* YJ56, KF212463 for *Arthrobacter ginsengisoli* DCY81, AF235091 for *Pseudarthrobacter sulfonivorans* ALL, X80741 for *Pseudarthrobacter polychromogenes* DSM20136, X83408 for *Pseudarthrobacter oxydans* DSM20119, AF330692 for *Pseudarthrobacter scleromae* YH-2001, KF150696 for *Arthrobacter bambusae* GM18, X80743 for *Paenarthrobacter nicotinovorans* DSM420, X83406 for *Paenarthrobacter histidinolovorans* DSM20115, BJMD01000050 for *Paenarthrobacter aurescens* NBRC12136, AJ512504 for *Paenarthrobacter nitroguajacolicus* G2-1, CAQI01000001 for *Pseudarthrobacter siccitolerans* 4J27, CP002379 for *Pseudarthrobacter phenanthrenivorans* Sphe3, CP001341 for *Pseudarthrobacter chlorophenolicus* A6, LT629779 for *Pseudarthroncter equi* IMMIBL-1606, AM409361 for *Pseudarthrobacter defluvii* 4C1-a, AB248526 for *Pseudarthrobacter niigatensis* LC4, MK211245 for *Pseudarthrobacter sp.* T11b, JF421614 for *Pseudarthrobacter enclensis* NIO-1008, AOFD01000111 for *Arthrobacter nitrophenolicus* SJCon, KM507333 for *Arthrobacter pokkalii* P3B162, KJ082091 for *Arthrobacter liuii* DSXY973, X80740 for *Arthrobacter pascens* DSM20545, BAEG01000072 for *Arthrobacter globiformis* NBRC12137, AB279890 for *Arthrobacter humicola* KV-653, AB279889 for *Arthrobacter oryzae* KV-651, NZ_CP080382 *Arthrobacter sp* PAMC25284, GCF_90009975.1 for *Arthrobacter cupressi* CGMCC1.10783, MH063435 for *Arthrobacter celericrescens* NEAU-SA2, BCQN01000021 for *Arthrobacter woluwensis* NBRC107840, KF479547 for *Arthrobacter nanjingensis* A33, KP128918 for *Arthrobacter ginkgonis* SYP-A7299, LC065376 for *Zafaria cholistanensis* NCCP-1664, AB778264 for *Zhihengliuella salsuginis* NBRC109062, BJNY01000040 for *Glutamicibacter uratoxydans* NBRC15515, NZ_FPCG00000000 for *Micrococcus terrus* CGMCC1.7054, GU797177 for *Citicoccus nitrophenolic* PNP1, AJ344143 *for Citricoccus muralis* 4–0. NC_002516.2 for *Pseudomonas aeruginosa*, NZ_CP026085.1 for *Escherichia coli* DH5*α*, NZ_012660.1 for *Pseudomonas fluorescens* SBW25, NC_018081.1 for *Enterococcus hirae* ATTC 9790, NC_000962.3 for *Mycobacterium tuberculosis* H37Rv. Similarly the protein dataset analyzed during the current study are available in the Protein Databank, Protein structure numbers: 1ZPUA for Iron transport multi-copper oxidase *Saccharomyces cerevisiae*, 1KYAA for Laccase *Trametes versicolor*, 1ASQA for Ascorbate oxidase *Cucurbita pepo*, 6KLGA for Laccase *Zea mays*, Q00292 for Dihydrogeodin oxidase *Aspergillus terreus*, 1KCWA for Ceruloplasmin *Homo sapiens*, NP001354161.2 for Hephaestin *Homo sapiens*, Q47452 for Copper resistance protein A *Escherichia coli*, 5B7MA for Blue copper oxidase *Escherichia coli K-12*, 2XLLA for Bilirubin oxidase *Albifimbria verrucaria*, 5ZLMA for Spore coat protein A *Bacillus subtilis* subsp. *Subtilis* str. 168, 3GYRA for Phenoxazinone synthase *Streptomyces antibiotics*, 1AS6A for Nitrite reductase *Alcaligenes faecalis*, 1Z2GA for Cytochrome c oxidase *Saccharomyces cerevisiae*, 1IC0A for Nitrosocyanin *Nitrosomonas europaea*, 7AQAA for Nitrous oxide reductase *Pseudomonas stutzeri*, 1BXAA for Amicyanin *Paracoccus denitrificans*, 1RCYA for Rusticyanin *Acidithiobacillus ferrooxidans*, 1XB6A for Azurin *Pseudomonas aeruginosa*, 1QHQA for Auracyanin *Chloroflexus aurantiacus*, 9PCYA for Plastocyanin *Phaselus vulgaris*, 3ERXA for Pseudoazurin *Paracoccus pantotrophus*, AAF92880 for EpoA *Sorangium cellulosum* SMP44, P39442 for Halocyanin *Natronomonas pharaonis*, IF56 A for Plantacyanin *Spinacia oleracea*, 1WS8B for Mavicyanin *Cucurbita pepo*, 1JERA for Stellacyanin *Cucumis sativus*, and 1X9UA for Umecyanin *Armoracia rusticana*.

## Supplementary Materials

Supplementary Materials Supplementary Materials The matching results of EzBioCloud database, multi-copper oxidase domain protein gene clusters in the SEED database and protein sequences similarity for the strain PAMC25284 are shown in Tables S1, S2, and S3, respectively.
