# Supplementary material for: Complete Genome Sequencing of Polar Arthrobacter sp. PAMC25284, Copper Tolerance Potential Unraveled with Genomic Analysis
Source: Int J Microbiol. 2022 Aug 25;2022:1162938. doi: 10.1155/2022/1162938 (PMC9436591; doi:10.1155/2022/1162938)
Supplement: Supplementary Materials — The matching results of EzBioCloud database, multi-copper oxidase domain protein gene clusters in the SEED database and protein sequences similarity for the strain PAMC25284 are shown in Tables S1, S2, and S3, respectively. [file 1162938.f1.docx]

**Complete genome sequencing of polar *Arthrobacter* sp. PAMC25284, copper tolerance potential unraveled with genomic analysis**

Jayram Karmacharya^1^, Prasansah Shrestha^1^, So-Ra Han^1^, Hyun Park^2^ and Tae-Jin Oh^1,3,4,*^

**Supplementary Materials**

**Table S1**. The results of EZBiocloud database matching for the strain PAMC25284.

| **Species (Hit taxon)** | **ANI (%)** | **ANI coverage (%)** | **Gene identity (%)*** |
| --- | --- | --- | --- |
| *Arthrobacter oryzae* KV-651 | 99.75 | 97.16 | 99.79 |
| *Arthrobacter humicola* KV-653 | 99.20 | 97.35 | 99.45 |
| *Arthrobacter globiformis* NBRC12137 | 97.35 | 97.25 | 98.48 |
| *Pseudoarthrobacter siccitolerans* 4J27 | 98.80 | 95.29 | 98.27 |
| *Arthrobacter pascens* DSM20545 | 98.55 | 97.25 | 99.17 |

Abbreviation: ANI, average nucleotide identity

*16s rRNA gene identity obtained from the Ezbiocloud database

**Table S2**. The gene clusters of multicopper oxidase domain containing protein based on annotations in the SEED database within the genome of *Arthrobacter* sp. PAMC25284.

| **Feature ID** | **Type** | **contig** | **Start** | **Stop** | **Length (bp)** | **Funcion** | **Subsystems** |
| --- | --- | --- | --- | --- | --- | --- | --- |
| fig\|2861279.3.peg468 | CDS | NZ_CP080382.1 | 476536 | 476985 | 450 | Multicoppper oxidase | Copper homeostasis |
| fig\|2861279.3.peg759 | CDS | NZ_CP080382.1 | 769267 | 770733 | 1,467 | Multicoppper oxidase | Copper homeostasis |
| fig\|2861279.3.peg866 | CDS | NZ_CP080382.1 | 861931 | 863529 | 1,599 | Multicopper oxidase | Copper homeostasis |
| fig\|2861279.3.peg887 | CDS | NZ_CP080382.1 | 876791 | 878326 | 1,536 | Multicopper oxidase | Copper homeostasis |
| fig\|2861279.3.peg1600 | CDS | NZ_CP080382.1 | 1628137 | 1628304 | 168 | Multicopper oxidase | Copper homeostasis |
| fig\|2861279.3.peg319 | CDS | NZ_CP080382.1 | 320084 | 321697 | 1,614 | Laccase (EC 1.10.3.2) | None |
| fig\|2861279.3.peg469 | CDS | NZ_CP080382.1 | 477017 | 478267 | 1,251 | Laccase (EC 1.10.3.2) | None |

**Table S3**. The sequence similarity of proteins of *Arthrobacter* sp. PAMC25284 with proteins of respective reference strains. The value was determined by the protein blast (blastp) tool of NCBI. ̶ , not applicable; NS, non-similarity.

|  | **Sequence similarity (%)** | | | | | | | | | |  |
| --- | --- | --- | --- | --- | --- | --- | --- | --- | --- | --- | --- |
| *Arthrobacter* sp. PAMC25284 | *E. hirae* ATCC9790 | | *M. tuberculosis* H37Rv | *P. aeruginosa* PAO1 | | | *E. coli* DH5α | | *P. fluorescens* SBW25 | |  |
| **P-type copper ATPase (P-type)** | **CopA** | **CopB** |  | **CopA1** | | **CopA2** | | **CopA** | |  | |
| P-type ATPase (KY499_RS03705) | 35.58 | 43.90 | ̶ | NS | | NS | | 35.51 | | ̶ | |
| P-type ATPase (KY499_RS04025) | 41.66 | 30.08 | ̶ | NS | | NS | | 36.59 | | ̶ | |
| P-type ATPase (KY499_RS04195) | 35.58 | 43.90 | ̶ | NS | | NS | | 35.51 | | ̶ | |
| P-type ATPase (KY499_RS10680) | 28.83 | 28.29 | ̶ | NS | | NS | | 32.70 | | ̶ | |
| P-type ATPase (KY499_RS12155) | 40.87 | 29.46 | ̶ | NS | | NS | | 38.39 | | ̶ | |
| **TatABC translocation system (TatABC)** | | | | | | | | | | |  |
| tatA (KY499_RS02890) | ̶ | | 50.00 | 28.57 | | | 29.87 | | ̶ | |  |
| tatB (KY499_RS16450) | ̶ | | 27.05 | 31.58 | | | 33.93 | | ̶ | |  |
| tatC (KY499_RS02895) | ̶ | | 35.64 | 34.39 | | | 33.07 | | ̶ | |  |
| **Cytoplasmic copper chaperone (CYTO-C)** | | | | | | | | | | |  |
| copZ (KY499_RS112160) | 32.26 | | ̶ | 35.94 | | | ̶ | | ̶ | |  |
| **Transcription factor (TF)** | | | | | | | | | | |  |
| MerR (KY499_RS05070) | ̶ | | ̶ | 26.98 | | | 24.14 | | ̶ | |  |
| MerR1 (KY499_RS05080) | ̶ | | ̶ | 34.55 | | | 36.36 | | ̶ | |  |
| MerR2 (KY499_RS11345) | ̶ | | ̶ | 39.13 | | | 35.71 | | ̶ | |  |
| MerR3 (KY499_RS13545) | ̶ | | ̶ | 34.78 | | | 37.68 | | ̶ | |  |
| MerR4 (KY499_RS14100) | ̶ | | ̶ | NS | | | 33.75 | | ̶ | |  |
| **Multicopper oxidase (MCO)** |  | | **MMCo** | **PcoA** | **PcoB** | | **CueO** | |  | |  |
| Mco1 (KY499_RS02210) | ̶ | | 22.76 | 24.55 | NS | | 28.29 | | ̶ | |  |
| Mco2 (KY499_RS04160) | ̶ | | 25.94 | 27.90 | NS | | 28.88 | | ̶ | |  |
| Mco3 (KY499_RS01525) | ̶ | | 24.69 | 36.05 | NS | | 25.95 | | ̶ | |  |
| Mco4 (KY499_RS03595) | ̶ | | 27.89 | 31.21 | NS | | 29.84 | | ̶ | |  |
| Mco5 (KY499_RS04055) | ̶ | | 40.61 | 28.05 | 40.00 | | 34.85 | | ̶ | |  |
| **Copper uptake system (CU)** | | | | | | | | | | |  |
| CopD (PFLU_RS19275) | ̶ | | ̶ | ̶ | | | ̶ | | ̶ | |  |
| CopC (KY499_RS10535) | ̶ | | ̶ | ̶ | | | 28.32 | | 34.96 | |  |
